# Chronic upregulation of circadian clock protein per1 among OSA patients

**DOI:** 10.1192/j.eurpsy.2021.1479

**Published:** 2021-08-13

**Authors:** A. Gabryelska, M. Sochal, P. Bialasiewicz

**Affiliations:** Department Of Sleep Medicine And Metabolic Disorders, Medical University of Lodz, Poland, Łódź, Poland

**Keywords:** OSA, circadian clock, PER1, PSG

## Abstract

**Introduction:**

PER1 is a repressor protein involved in regulating circadian rhythm. While obstructive sleep apnea (OSA) is characterized by recurred pauses in breathing caused by the collapse of the upper airways it might be associated with disruption of the circadian clock.

**Objectives:**

The study aimed to assess PER1 protein in OSA patients and evaluate its association with PSG parameters.

**Methods:**

The study included 40 individuals, who underwent diagnostic polysomnography (PSG) examination. Based apnea-hypopnea index (AHI) patients were divided into groups: control (AHI<5; n=10) and OSA (AHI5; n=30). All participants had their peripheral blood collected in the evening (9:00-10:00 pm) before and in the morning (6:00-7:00 am) after the PSG. PER1 protein concertation measurements were performed using ELISA. Funding: National Science Centre, Poland-2018/31/N/NZ5/03931.

**Results:**

The control and OSA group were match in sex and age, while differed regarding BMI (p=0.039), desaturation index (p<0.001) and AHI (p<0.001). PER1 protein level was elevated in OSA group compared to control both in the evening (322.384.1vs.208.460.1pg/ml;p<0.001) and morning (314.891.9vs.228.157.3pg/ml;p=0.002). No difference was observed between evening and morning PER1 level (p=0.946). Morning PER1 correlated with AHI (r=0.400; p=0.011), desaturation index (r=0.391;p=0.013), age (r=-0.312;p=0.049) and BMI (r=0.383;p=0.015). In a multiple linear regression model (R^2^=0.268;p=0.003) morning PER1 protein level was influenced by age (p=0.006) and AHI (p=0.025), while BMI and desaturation index were not significant.

**Conclusions:**

OSA patients might suffer from circadian clock disruption, which is mainly associated with the severity of the disorder and age. Further studies are needed as this dysregulation can result in metabolic and mood disorders often observed in this group of patients.

